# Linking Air Pollution and Hematologic Malignancies: From Epidemiology to Molecular Mechanisms

**DOI:** 10.1007/s11899-026-00785-2

**Published:** 2026-08-11

**Authors:** Hamsa Murli, Sadia Alam, Evelyn Mesler, Aditi Shastri

**Affiliations:** 1 Albert Einstein College of Medicine, Bronx, NY USA; 2 Department of Internal Medicine, Jacobi Medical Center, NYC Health and Hospitals, Bronx, NY USA; 3 Department of Internal Medicine, Montefiore Medical Center, Bronx, NY USA; 4 Division of Hematologic Malignancies, Department of Oncology, Einstein Comprehensive Cancer Center, Montefiore, Bronx, NY USA

**Keywords:** Outdoor air pollution, Hematologic malignancies, Leukemia, Lymphoma, Benzene, Particulate matter

## Abstract

**Purpose of Review:**

Outdoor air pollution (OAP) is a major global environmental health threat associated with respiratory disease and solid tumors, with growing evidence linking it to hematologic malignancies. This review summarizes the molecular mechanisms and epidemiologic evidence connecting OAP exposure to acute and chronic leukemias, lymphomas, myelodysplastic syndromes (MDS), clonal hematopoiesis of indeterminate potential (CHIP), and multiple myeloma (MM).

**Recent Findings:**

Pollutants implicated in hematologic carcinogenesis include particulate matter (PM), benzene, nitrogen dioxide (NO₂), sulfur dioxide (SO₂), arsenic, and ethylene oxide. Molecular mechanisms include oxidative stress, DNA damage, epigenetic dysregulation, chronic inflammation, and hematopoietic stem cell dysfunction. Epidemiologic evidence is strongest for acute myeloid leukemia (AML), particularly with benzene exposure. Acute lymphoblastic leukemia (ALL) has also been associated with traffic-related pollution, NO₂, and PM exposure, though findings are less consistent. Certain lymphoma subtypes demonstrate pollutant-specific associations, while emerging evidence suggests possible links between OAP exposure and MDS and CHIP. In contrast, evidence for chronic leukemias and MM remains limited.

**Summary:**

Despite limitations, including exposure misclassification and residual confounding, cumulative molecular and epidemiologic evidence supports OAP as an important and potentially modifiable risk factor for hematologic malignancies. OAP exposure disproportionately affects racial and ethnic minorities, low-income communities, and rapidly industrializing regions, magnifying existing health disparities. Further research, environmental policy reform, and public health interventions are needed to reduce exposure and disease burden.

**Graphical Abstract:**

HSC: hematopoietic stem cell. Created in BioRender. Murli, H. (2026) https://BioRender.com/tkhy5k7

**Figure:**
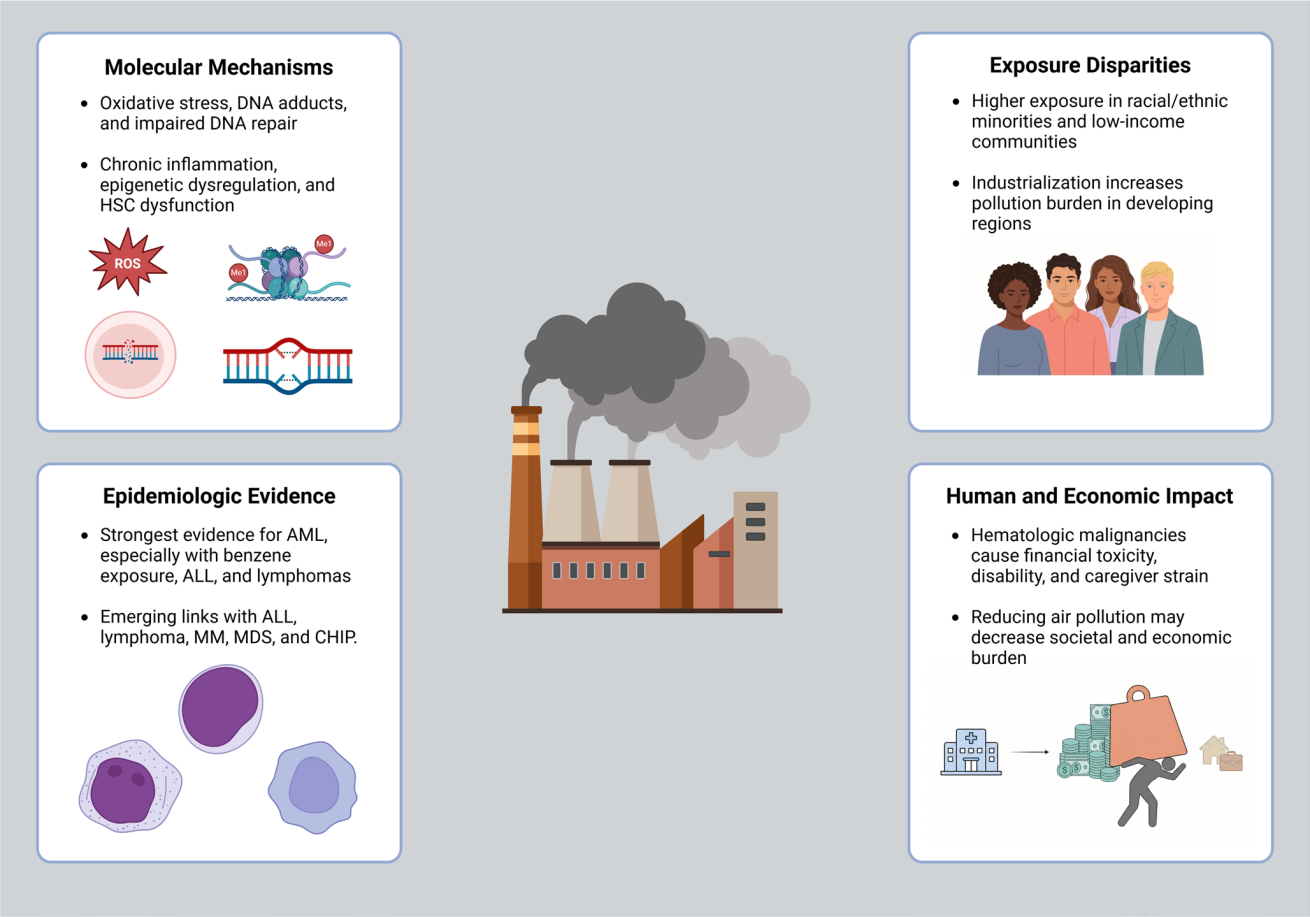

## Introduction

Hematologic malignancies represent a growing global health burden, with incidence steadily increasing in recent decades and projected to continue rising [1, 2]. This trend is partly driven by aging populations and improved diagnostics; nevertheless, it also reflects an increase in the true incidence of hematologic malignancies. This growing burden is especially concerning in lower-income countries, where the incidence is rising steeply despite limited access to treatment modalities. Beyond mortality, this leads to a significantly worse quality of life [3], financial toxicity [4, 5], and disability [6]. The pathogenesis of hematologic malignancies remains an enigma, underscoring the need to better characterize potentially modifiable environmental contributors.

Air pollution is defined as the presence of substances in the air at concentrations or durations higher than naturally expected, with the potential to adversely affect health [7]. Outdoor Air Pollution (OAP) consists of a combination of liquid, gaseous, and solid particles suspended in the atmosphere. This mixture includes pollutants like carbon monoxide (CO), lead (Pb), nitrogen dioxide (NO_2_), ozone (O_3_), particulate matter (PM), and sulfur dioxide (SO_2_) [8]. Given this heterogenous mixture of pollutants, each with distinct toxicologic properties, associations observed with overall OAP exposure may reflect the contribution of the individual components and correlated exposures rather than a uniform biologic effect. OAP is well known for its association with respiratory disorders and lung cancer; however, it has far-reaching health consequences involving multiple organ systems [9]. According to the 2021 Global Burden of Disease (GBD) [10] data, particulate matter air pollution was responsible for 8% of total disability-adjusted life years (DALY) lost, making it the leading contributor to GBD, surpassing other risk factors like smoking, hypertension, and diabetes. It is now considered one of the greatest environmental threats to human health, with 13 attributed deaths every minute worldwide [11]. Ambient (outdoor) air pollution led to 4.2 million premature deaths in 2019, 89% of which were in low and middle-income countries. When accounting for household (indoor) air pollution, the number totals to 6.7 million [12].

An association between environmental pollutants and cancer has been debated since the 1950s, when many studies showed an urban-rural divide in lung cancer incidence, even after accounting for cigarette smoking [13]. It was officially classified as a Group 1 carcinogen by the International Agency for Research on Cancer (IARC) in 2013 [14]. Among solid tumors, other reported associations are with genitourinary malignancies [15, 16], including bladder, kidney, and prostate cancers, breast cancer [17–19], and gastrointestinal malignancies [20, 21].

There is a growing body of evidence that this association may extend beyond solid tumors. In 2018, the IARC concluded that exposure to benzene was associated with an increase in the risk of Acute Myeloid Leukemia (AML), with limited but positive data for Acute Lymphoblastic Leukemia (ALL) and non-Hodgkin lymphomas (NHL) in adults [22]. A Danish case-control study found that NO_2_ increased the risk of AML in an adult population [23]. Another recent study by Diver et al. [24] found correlations between individual pollutants and specific types of lymphomas. OAP was associated with increased mortality from hematologic malignancies in a large cohort of US adults [25]. Other studies have shown mixed results [26–30], possibly owing to the heterogeneity of hematologic malignancies, with variable disease biology. To our knowledge, a single systematic review and dose-response meta-analysis has been completed on this topic by Lin et al. [31] in 2023, who reported sustained significant associations of OAP exposure with hematologic malignancies, modified by age, disease subtype, and geographic region.

With this review, we aim to provide an updated perspective on the epidemiologic evidence linking air pollution to the different subtypes of hematologic malignancies with a focus on the molecular mechanisms of carcinogenesis. We also address disparities, the human and economic toll of the rising burden of hematologic malignancies, as well as the importance of public policy in combating air pollution.

## Air Pollutants and Molecular Mechanisms of Carcinogenesis

### Particulate Matter (PM_2.5_ and PM_10_)

Exposure to airborne particulate matter (PM) contributes to carcinogenesis through multiple mechanisms of DNA damage. PM consists of airborne solid particles and liquid droplets stratified by size [32]. PM_10_ includes particles ≤ 10 μm in diameter, including dust, pollen, and mold, whereas PM_2.5_ refers to fine particulate matter < 2.5 μm derived from natural and anthropogenic sources such as industrial emissions and fossil fuel combustion. Their smaller size allows PM_2.5_ particles to penetrate the respiratory tract and enter systemic circulation [33, 34]. PM_2.5_ exposure is associated with increased reactive oxygen species (ROS) generation, oxidative DNA damage, and methylated DNA adduct formation [33, 34]. In human myeloid leukemia cells, low-dose PM2.5 exposure increased ROS levels, inflammatory markers including IL-6 and COX-2, inflammatory gene expression, and leukemia cell growth in vitro and in vivo [35]. Black carbon/PM_2.5_ exposure promotes IL-1β-mediated inflammation, alters hematopoietic progenitor dynamics, and enhances expansion of TET2-mutant clones, supporting a role in myeloid carcinogenesis [36].

Compared with PM_2.5_, PM_10_ exposure is associated with a greater burden of genomic alterations and DNA repair-related mutational signatures, whereas PM_2.5_ is more strongly associated with oxidative stress-related signatures [37]. Persistent oxidative stress and epigenetic dysregulation, including hypermethylation of tumor suppressor genes, contribute to malignant transformation in hematopoietic and other tissues [38].

### Benzene

Benzene is a well-established environmental and occupational toxin associated with bone marrow toxicity and leukemia risk [39]. It is primarily emitted from coal and oil industries, motor vehicle exhaust, and tobacco smoke [40]. Genomic analyses of benzene-exposed workers identified alterations in genes involved in immune signaling, DNA repair, and cell survival, including NFKB1, along with dose-dependent DNA methylation changes and altered miRNA regulation indicative of persistent epigenetic reprogramming [41]. Benzene-associated molecular signatures overlapped significantly with those of AML, suggesting a role in early leukemogenic changes in hematopoietic progenitor cells prior to overt malignancy [41]. Cytogenetic studies further demonstrated similarities between benzene-associated AML and de novo AML [42]. These findings support experimental data showing that benzene metabolites induce oxidative stress, DNA damage, and disruption of hematopoietic stem and progenitor cell function, leading to impaired hematopoiesis and clonal malignant transformation.

### Arsenic

Arsenic, an element emitted by high-temperature processes such as coal-fired power plants and burning vegetation [43], disrupts early hematopoiesis through multiple mechanisms. Medina et al. demonstrated that arsenite exposure impairs early hematopoiesis by reducing the binding capability of transcription factor GATA-2 to regulatory DNA elements, disrupting hematopoietic differentiation, and promoting accumulation of immature progenitor cells [44]. Chronic low-level arsenic exposure aberrantly activates the PI3K/Akt signaling pathway, promoting survival and proliferation of hematopoietic progenitors while impairing apoptotic checkpoints [45]. Interestingly, arsenic also serves as a therapeutic modality in acute promyelocytic leukemia, highlighting its paradoxical capacity to exert both pro- and anti-carcinogenic effects. Further studies are needed to better characterize the exposure contexts, molecular pathways, and biologic conditions underlying these divergent effects.

### Ethylene Oxide (EtO)

EtO, a Group 1 carcinogen emitted by industrial and sterilization sites, is a direct alkylating agent with a well-characterized genotoxic mechanism [46]. Walker et al. demonstrated in murine models that inhaled EtO forms covalent DNA adducts in a dose-dependent manner across multiple tissues [47]. These adducts persist due to incomplete DNA repair, promoting mutational accumulation during replication. The continuous division of hematopoietic stem and progenitor cells likely increases their vulnerability to clonal expansion and malignant transformation. These mechanistic findings support epidemiologic evidence linking occupational EtO exposure to increased leukemia and lymphoma risk.

### Nitrogen Dioxide (NO_2_)

NO_2_ is primarily emitted through fossil fuel combustion from vehicles, power plants, industrial facilities, household appliances, and tobacco smoke [48]. NO₂ is a reactive free-radical gas that enters systemic circulation and generates ROS, overwhelming cellular antioxidant defenses, causing DNA strand breaks, DNA-protein crosslinks, and the formation of micronuclei in bone marrow cells [49]. NO₂ further impairs cellular function and promotes chronic inflammation through protein nitration (e.g., nitrotyrosine formation) [50]. Cumulative genotoxicity, persistent oxidative stress, and long-term immune dysregulation contribute to malignant cellular transformation. However, as NO_2_ is frequently studied alongside other pollutants such as PM_2.5_ and benzene, isolating NO_2_’s independent contribution to hematologic malignancy remains challenging.

### Sulfur Dioxide (SO_2_)

**Fig. 1:**
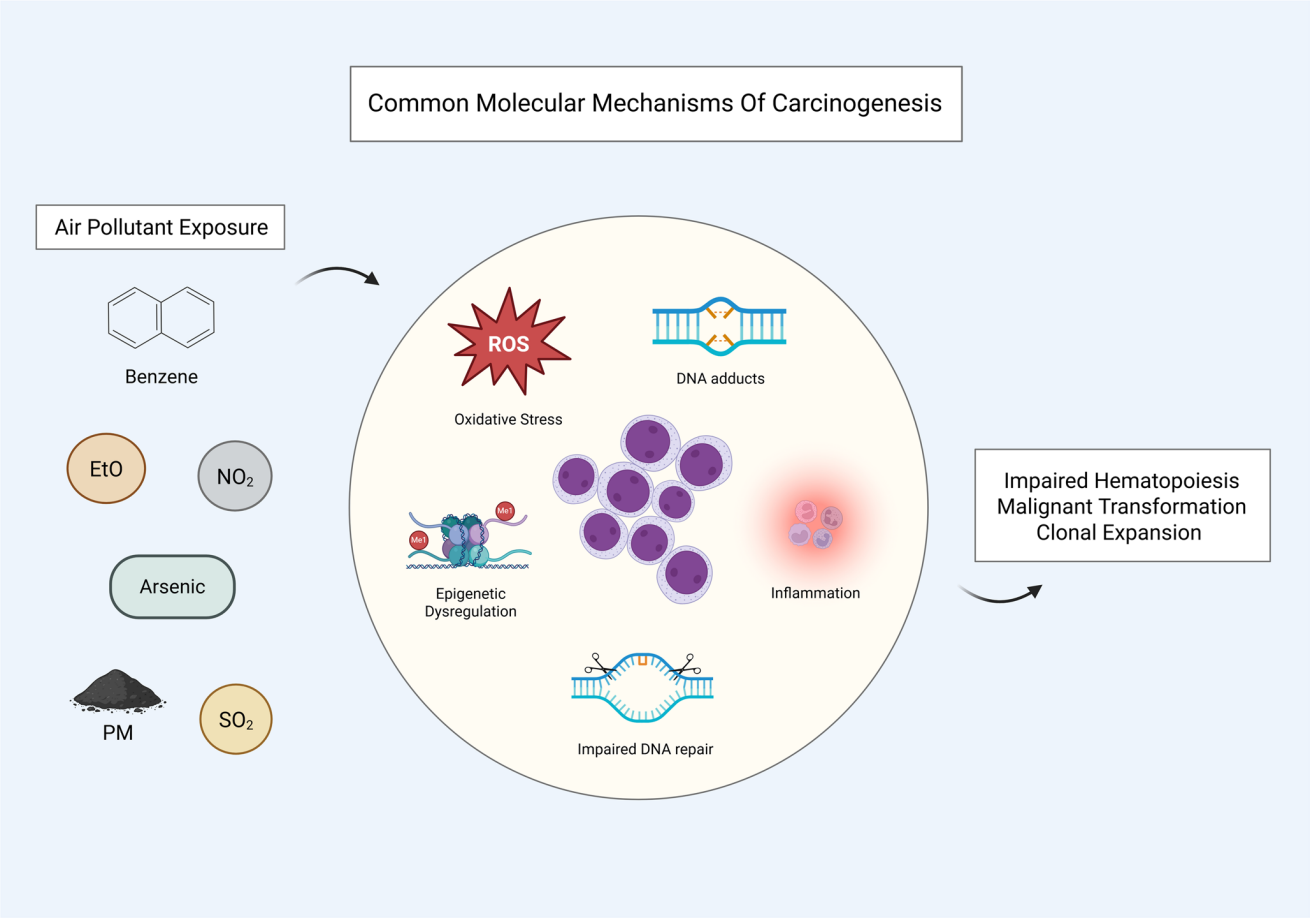
Molecular Mechanisms of Carcinogenesis. Created in BioRender. Murli, H. (2026) https://BioRender.com/tkhy5k7

SO_2_ is an air pollutant with significant carcinogenic potential. Upon inhalation, SO_2_ hydrates in the airways to form sulfurous acid, dissociating into bisulfite and sulfite ions that enter systemic circulation and distribute to multiple organs, including the bone marrow [51, 52]. SO_2_ is associated with direct DNA damage, as evidenced by a higher frequency of chromosome aberrations, sister-chromatid exchanges, and micronuclei formation across humans, murine, and in-vitro models [52–55]. It also acts as a co-carcinogen [56] by depleting cellular glutathione [57], enhancing pollutant-DNA binding [56], activating procarcinogens into their carcinogenic metabolites [58], and potentiating the mutagenicity of co-pollutants such as particulate matter [59]. Additionally, SO_2_ upregulates proto-oncogenes and downregulates tumor suppressor genes relevant to hematologic malignancies, including c-myc, p53, p16, and Rb [60]. Epidemiologically, SO_2_ exposure demonstrates a dose-response relationship with non-Hodgkin lymphoma (NHL) incidence and has been linked to increased mortality from leukemias and NHL among pulp and paper industry workers [61], though further research is needed to characterize its effects at ambient exposure levels on the general population (Fig. 1).

## Epidemiological Evidence Linking Air Pollution and Hematologic Malignancies

A landmark cohort of Pliofilm rubber workers provided foundational epidemiologic evidence establishing benzene as a leukemogen, particularly in relation to acute myeloid leukemia (AML) [62, 63]. While early studies focused on high-dose occupational exposure, subsequent research has increasingly explored lower-level environmental exposure in the general population [64, 65]. Occupational cohort studies demonstrated hematopoietic toxicity, including cytopenias, even at benzene exposures below 1 ppm, supporting leukemogenic potential at relatively low exposure levels [66]. Although lifetime exposures below 50 ppm-years were initially considered unlikely to significantly increase AML risk [67], later studies demonstrated elevated AML risk at substantially lower exposure levels, including among petrochemical workers exposed to 2–8 ppm-years [68]. Additional studies have reported leukemia risk following prolonged exposure to gasoline vapors after environmental spills, as well as dose-response relationships between cumulative benzene exposure and hematologic malignancies [69]. More recently, multiple population-based studies have demonstrated increased AML incidence in communities with higher ambient benzene exposure [70]. Collectively, these findings suggest leukemogenic potential even at lower environmental exposure levels.

The strength of epidemiologic evidence linking outdoor air pollution to hematologic malignancies varies considerably across disease entities. While the association between benzene exposure and AML is supported by extensive occupational, environmental, and mechanistic evidence, associations involving MDS, CHIP, and specific lymphoma subtypes are supported by a smaller but growing body of literature. In contrast, evidence linking ambient air pollution to chronic leukemias and multiple myeloma remains limited and inconsistent. Accordingly, the epidemiologic literature is discussed below according to the strength of the available evidence rather than by disease category alone (Table 1).

**Table 1:** Strength of epidemiologic evidence linking outdoor air pollution to hematologic malignancies

Strength of Evidence	Hematologic Malignancy	Pollutant(s)	Summary of Evidence
Strong	AML	Benzene	Consistent occupational, environmental, dose-response, cohort, and meta-analytic evidence supports the strongest epidemiological association
	Childhood acute leukemias	Traffic-related pollution	Multiple studies and a meta-analysis demonstrate increased risk, although individual pollutants are less consistent
Emerging	ALL	Benzene, PM_2.5_, NO_2_	Evidence suggests increased risk, particularly in childhood disease, although results are heterogeneous across pollutants.
	Lymphomas	PM, NO_2_, CO, Benzene	Associations appear subtype-specific (mantle cell, marginal zone, Hodgkin lymphoma) rather than consistent across all lymphomas.
	MDS	Benzene, PM_2.5_	Several occupational and recent population and experimental studies suggest increased risk, but the literature remains relatively limited.
	CHIP	PM_2.5_	Multiple recent studies suggest increased CHIP prevalence and clonal burden, although the relationship with subsequent hematologic malignancy remains uncertain.
Limited or Uncertain	CLL	PM_2.5_, Benzene, 1,3-butadiene	Findings are inconsistent, with predominantly null meta-analytic evidence.
	CML	Benzene	Few studies available; evidence remains insufficient.
	Multiple Myeloma	PM_2.5_, NO_2_, O_3_	Incidence studies largely negative, although PM exposure may adversely affect survival after diagnosis.

### Strong Epidemiologic Evidence

#### Benzene and AML

With advances in exposure assessment methods, individual pollutants have increasingly been investigated. Among them, benzene demonstrates the strongest association with AML. A case-control study in Oklahoma found that children with AML had approximately threefold higher benzene exposure than controls [71], while prenatal benzene exposure has also been associated with increased childhood AML risk [72]. Lin et al. reported a pooled relative risk of 1.84 for AML with benzene exposure [31]. Although many studies combine occupational, household, and ambient exposures, limiting isolation of environmental effects alone [73], benzene exposure demonstrates a dose-dependent relationship with both AML and ALL, with stronger and more consistent associations observed for AML [73, 74].

#### Traffic-related Pollution and Acute Leukemias

OAP has increasingly been associated with acute leukemias, although the magnitude of risk appears to vary according to pollutant composition, exposure setting, and critical windows of exposure. Early epidemiologic studies used traffic density as a surrogate marker for OAP exposure and consistently demonstrated elevated leukemia risk in children living near high-traffic areas. In the French GEOCAP study, proximity to high-traffic roads was associated with a 1.2-fold higher risk of AML [75], while a Spanish study reported a threefold increase in leukemia risk [76]. Meta-analyses have since confirmed a modest but significant association between traffic-related pollution and acute leukemia risk [31, 74]. Collectively, epidemiologic studies and meta-analyses support a consistent association between traffic-related air pollution and childhood acute leukemias, although the specific pollutant(s) responsible remain less clearly defined than for benzene-associated AML.

### Emerging Epidemiologic Evidence

#### NO_2_ and Acute Leukemias

The epidemiologic evidence for NO₂ and particulate matter is comparatively less consistent. Some studies have demonstrated stronger associations between NO₂ exposure and ALL, particularly at exposure levels exceeding 40 µg/m³, whereas others reported stronger associations with AML [77, 78]. Prenatal exposure to nitrogen oxides has also been associated with increased ALL risk [79]. Meta-analyses have not consistently demonstrated significant associations between NO₂ exposure and acute leukemia overall, although dose-response relationships and regional variation, with over two-fold increase in acute leukemia risk in Asian populations, have been reported [31].

#### PM and Acute Leukemias

PM_2.5_ exposure demonstrates heterogeneous associations with acute leukemias [31]. Prenatal exposure to PM, particularly PM_10_, was associated with increased childhood leukemia risk in a Spanish study [80]. A Danish registry study found increased leukemia risk with PM_2.5_ exposure over 1-, 5-, and 10-year exposure periods, with the strongest effect observed in adults older than 70 years [81]. PM_2.5_ exposure in Denmark remained associated with increased leukemia odds even in multi-pollutant models [29]. However, other studies have reported null associations [82].

Several studies suggest a stronger relationship between PM exposure and ALL. A Texas study demonstrated increased ALL incidence in children living in counties with PM_2.5_ concentrations exceeding Environmental Protection Agency (EPA) standards, particularly among Hispanic children [83]. A Mexican study similarly identified clusters of increased ALL incidence correlating with higher PM_2.5_ levels [84]. In contrast, fewer studies have specifically examined AML, with the Danish registry study remaining among the most notable positive studies [81]. Despite these findings, the Lin et al. meta-analysis did not demonstrate statistically significant associations between PM exposure and either ALL or AML [31]. Additional pollutants, including 1,3-butadiene and ozone, have also demonstrated possible associations with childhood leukemia risk in isolated studies [85, 86].

#### Lymphomas

The US-based study by Diver et al. remains one of the more robust analyses of OAP and lymphoma risk. Higher PM_10−2.5_ concentrations were associated with increased risk of mantle cell lymphoma (HR 1.43 per 4.1 µg/m^3^ increase) [24]. Hodgkin lymphoma (HL) was associated with NO_2_ exposure, T-cell lymphoma with CO exposure, and marginal zone lymphoma with both NO_2_ and CO exposure, with hazard ratios of approximately 1.3 [24]. However, findings across lymphoma subtypes remain inconsistent. A large population-based study found no significant associations between multiple pollutants, including PM_2.5_, O_3_, SO_2_, and NO_2_, and NHL overall [87], although some pollutants demonstrated non-significant elevations in follicular lymphoma risk. Other studies similarly found no association between OAP and HL or NHL overall, though PM constituents were associated with follicular lymphoma in some analyses [30, 87, 88].

Prenatal exposure to NO_2_ and benzene has also been associated with increased HL risk, with one study demonstrating a significant dose-response relationship for NO_2_ exposure [89]. Additional environmental exposures linked to lymphoma risk include proximity to paper industries, solid waste incinerators, and dioxin exposure [90–92]. Overall, meta-analytic data demonstrate predominantly null associations between most pollutants and lymphoma risk, although notable exceptions include strong associations between benzene exposure and HL, as well as between PM_2.5_ exposure and adolescent NHL [31].

#### MDS

Vicinity to industrial plants and lifetime oil exposure are linked to a higher risk of MDS, along with specific occupations [93]. Low-level benzene exposure among petroleum workers demonstrated a monotonic dose-response relationship with MDS, notably without a corresponding association with AML, suggesting that MDS may be a more relevant entity at lower exposure levels, such as those encountered in OAP [94]. Occupational and environmental exposures are associated with abnormal cytogenetics in MDS [95]. More recently, long-term PM_2.5_ exposure was associated with increased incident MDS risk and higher-risk MDS phenotypes in a Bronx-based study [36].

#### Clonal Hematopoiesis of Indeterminate Potential

The most comprehensive study on Clonal Hematopoiesis of Indeterminate Potential (CHIP) and OAP comes from the 2024 study by Aboshahem et al., involving genotyping of over 7000 patients, which found not only an increased risk of CHIP with higher PM_2.5_ concentrations, but also an increased number of CHIP mutations. PM_2.5_ exposure also increased the risk of major cardiovascular events in patients with CHIP mutations [96]. The landmark study by Jasra et al., following first responders involved in the World Trade Center (WTC) tragedy, found increased rates of CHIP mutations with an odds ratio of over 3, in cases that were exposed to WTC PM, compared to controls [97]. Somatic mutations in DNMT3A and TET2 were increased. In contrast, a 2023 epidemiologic study found a positive but non-significant association between OAP, specifically PM_2.5_ and NO_2_, and risk of CHIP, likely limited by low power [98]. It is important to note that the association of pollution-associated CHIP and hematologic malignancy remains uncertain, especially given strong lifestyle factors such as age, smoking, and occupational hazards increasing inflammation. However, these findings do suggest that exposure to environmental toxins and gases can potentially influence early clonal evolution and CHIP.

### Limited or Uncertain Epidemiologic Evidence

#### Chronic Leukemias

Evidence linking OAP to chronic leukemias remains limited and less consistent than for acute leukemias. The Danish registry study by Puett et al. demonstrated an increased risk of CLL with higher 1-year average PM_2.5_ exposure [81]. In contrast, a Canadian case-control study found no significant associations between NO_2_ or PM_2.5_ exposure and CLL [28]. Although an inverse relationship was observed, this finding is unlikely to be causal, given the biologic implausibility, and may instead reflect residual confounding or underdiagnosis of an indolent malignancy in lower-resource, high-exposure regions.

Evidence linking benzene exposure to chronic leukemias is similarly limited. Older occupational case reports suggested possible associations [99], but subsequent occupational case-control studies did not demonstrate significant associations between benzene exposure and either CLL or CML [100]. Given that occupational exposures are typically substantially higher than environmental exposures, these findings may argue against a strong association between ambient benzene exposure and chronic leukemias. In contrast, 1,3-butadiene has shown more consistent associations with lymphoid malignancies, particularly CLL [101].

Meta-analytic data remain limited by the small number of available studies. Lin et al. were unable to generate pooled risk estimates for several pollutants in CLL because of insufficient data, while pooled estimates for NO_2_, PM_2.5_, and O_3_ were not statistically significant [31]. Similar limitations exist for CML, where epidemiologic evidence remains largely restricted to occupational studies [31, 102]. Although meta-analyses have not demonstrated significant associations, some studies suggest stronger associations in higher-quality and more recent cohorts [102].

#### Multiple Myeloma

A large European study with over 200,000 participants showed no association between OAP and the risk of MM. Pollutants studied included NO_2_, PM_2.5_, black carbon, and O_3_ [103]. However, the number of years of PM_2.5_ exposure above EPA and Word Health Organization standards was associated with lower overall survival in MM patients in a study involving over 1500 participants [104]. Self-reported exposure to CO, along with other toxic compounds, was found to increase the risk of MM in another study; however, occupational exposures were included [105]. Diesel exhaust was not associated with elevated MM risk based on a review of existing studies [106]. Meta-analysis was limited by meager data, but found null associations for all pollutants studied [31].

## Disparities, Societal Burden, and Public Health Implications

OAP is not a homogenously distributed risk factor, and there is considerable variation in exposure rates globally, within countries, and even within individual cities. In the United States, significant geographic disparities in OAP exposure persist despite overall improvements in air quality over recent decades [107]. These disparities are inextricably linked to socioeconomic and racial factors, with racial and ethnic minorities disproportionately more likely to live near industrial plants and hazardous waste incinerators [108, 109]. Analysis of PM_2.5_ exposure data in the United States from 2000 to 2016 demonstrated consistently higher concentrations in communities with larger Black, Hispanic, and Asian populations, whereas predominantly White neighborhoods generally experienced lower exposure levels [110]. Low-income communities may also face modestly higher pollutant exposure burdens [109, 110].

In recent years, OAP has emerged as a crucial concern in rapidly developing countries [111]. Industrialization comes with its environmental costs, especially when a conscious investment is not made into sustainable energy sources. The chemical constituents of OAP may be vastly different in developing countries, leading to varying health effects. Indoor air pollution due to traditional cooking fuels also becomes a much larger consideration compared to developed countries [112]. There is a substantial need for more OAP research in these countries to identify and combat unique challenges.

As the global burden of hematologic malignancies continues to rise, so do their individual and societal consequences. Beyond morbidity and mortality, these diseases impose substantial direct and indirect financial burdens on patients, caregivers, and healthcare systems. High insurance-related expenses and out-of-pocket (OOP) costs contribute significantly to financial hardship, particularly in acute myeloid leukemia (AML), where annual OOP costs may exceed $7,000 [113]. Although advances such as chimeric antigen receptor T-cell (CAR-T) therapy and allogeneic hematopoietic stem cell transplantation (HSCT) have improved outcomes, they often further increase long-term financial strain [114–116]. Patients also experience major indirect costs, including loss of productivity and disability after treatment [117]. Collectively, these economic and psychosocial consequences have been termed the “financial toxicity” of cancer care [118].

The financial burden of hematologic malignancies also extends to caregivers, who frequently experience reduced productivity and employment disruptions while providing care [119]. Cancer survivors demonstrate lower long-term employment rates, contributing to substantial societal productivity losses [120, 121]. Hematologic malignancies additionally impose disproportionately high healthcare expenditures compared with many other cancers, driven in part by resource-intensive therapies such as allogeneic hematopoietic stem cell transplantation (HSCT) and chimeric antigen receptor T-cell (CAR-T) therapy [115, 122–124]. In a 2017 study, the cost of a single patient’s treatment across three years following an acute leukemia diagnosis was estimated to be over $800,000 USD, compared to $250,000 for lung cancer and under $150,000 for colorectal cancer [122]. Consequently, public health policies aimed at reducing air pollution may provide not only health benefits, but also meaningful economic benefits for patients, caregivers, healthcare systems, and society as a whole.

## Conclusion

Our review demonstrates strong mechanistic evidence supporting the biologic plausibility of OAP-induced carcinogenesis in hematologic malignancies. Although epidemiologic evidence varies across pollutants, exposure levels, and malignancy subtypes, the overall literature is highly suggestive of a significant association between OAP exposure and hematologic malignancies, particularly AML and benzene exposure. These relationships are further modified by socioeconomic and geographic disparities.

Important limitations remain in the current literature. Many studies evaluate leukemia broadly without adequately distinguishing among specific subtypes, despite substantial biologic and clinical heterogeneity. Existing epidemiologic data are largely ecological and population-based, making them susceptible to unmeasured confounding and exposure misclassification. Exposure assessment represents a particularly important limitation. Residential address is often used as a proxy for exposure, but ambient pollutant concentrations at the place of residence may not accurately reflect the entire exposome, which is also shaped by time spent at work, commuting patterns, indoor air quality, and other daily activities. Occupational exposures may exceed or otherwise confound the effects of ambient pollution, while indoor sources and building characteristics may either amplify or attenuate exposure. Residential mobility over time further complicates estimation of cumulative and latency-relevant exposures, particularly for malignancies that may develop over many years. Socioeconomic factors also influence where individuals live and work, housing quality, occupational risk, access to cleaner environments, and the likelihood of exposure to multiple pollutants, thereby contributing to exposure heterogeneity.

Older studies additionally relied on imprecise pollutant exposure models, such as EPA ASPEN [125], rather than direct pollutant measurements. Studies based on residential addresses are further limited by residential mobility over time. Moreover, the diagnostic classification of hematologic malignancies, particularly along the AML-MDS spectrum, continues to evolve, contributing to variability in outcome definitions across older and newer studies. Nonetheless, emerging evidence linking OAP exposure to entities such as MDS and CHIP highlights the need for continued mechanistic and population-based research.

The Clean Air Act was associated with 300 fewer leukemia deaths between 2020 and 2025 [126]. Although the effect of smoking cessation remains greater, reducing OAP represents an important complementary public health strategy given its widespread exposure and broad health consequences. Since OAP is a potentially modifiable environmental contributor that cannot be meaningfully addressed at the individual level alone, continued efforts from policymakers and healthcare systems are essential to improve air quality and reduce the burden of hematologic malignancies. Our role as healthcare providers is to continue researching the consequences of OAP, empower patients to advocate for cleaner air, and ourselves ardently advocate for better air quality for all.

## Data Availability

No datasets were generated or analysed during the current study.
